# Supplementary material for: Kinetic Analysis of Prostate-Specific Antigen Interaction with Monoclonal Antibodies for Development of a Magnetic Immunoassay Based on Nontransparent Fiber Structures
Source: Molecules. 2022 Nov 21;27(22):8077. doi: 10.3390/molecules27228077 (PMC9693269; doi:10.3390/molecules27228077)
Supplement: Supplementary file 1 [file molecules-27-08077-s001.zip › molecules-1997460-supplementary.pdf]

*Supplementary Materials*

## S1. Testing non-specific fPSA binding directly to the glass slide

The absence of non-specific contribution due to fPSA binding directly to the slide rather than to the antibody was verified in control experiments using the glass sensor chips without immobilized antibody to fPSA. Instead of antibodies, in these experiments we pumped along the sensor chip blocking buffer followed by passing fPSA antigen. No noticeable non-specific binding of fPSA with the glass surface was registered (Figure S1)

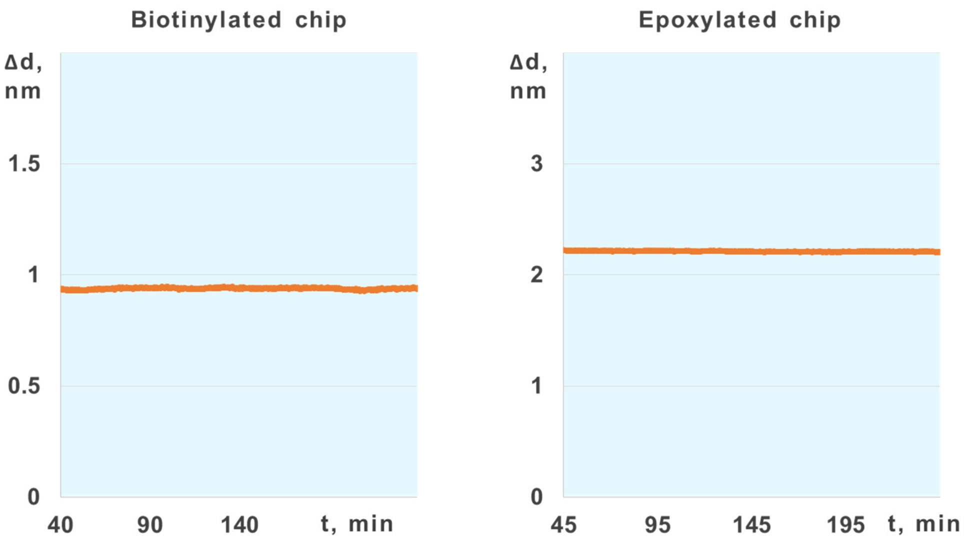


**Figure S1.** Testing non-specific fPSA binding directly to the glass slide

## S2. Specificity of unlabeled and biotinylated antibodies

Specificity characterization of unlabeled (4H3 clone) and biotinylated (ICO168 clone) antibodies was performed via testing the following non-target antigens in concentrations exceeding the physiological ranges:  carcinoembryonic antigen (CEA), 100 ng/mL; alpha-fetoprotein (AFP), 300 ng/mL; ovarian cancer-related tumor marker CA125, 150 U/mL; thyroid stimulating hormone (TSH), 300 mIU/L; triiodothyronine (T3), 5 ng/mL; thyroxine (T4), 200 pM. No noticeable changes in the biolayer thickness were registered at the stage of antigen binding (Figure S2). Thus, the studied antibodies demonstrated high specificity, probably because they were of commercial grade so that their manufacturers paid particular attention to selection of highly specific clones.

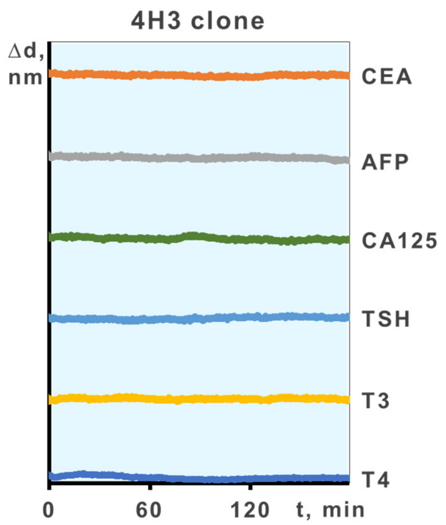


**Figure S2.** Characterization of specificity of unlabeled (4H3 clone) and biotinylated (ICO168 clone) antibodies

**S3. Testing epitopes of unlabeled 4H3 and biotinylated ICO168 antibodies for non-overlapping**

Antibodies were pre-tested with the label-free mSCI to check their effectiveness in forming the "antibody – antigen – antibody" immune sandwich (Figure S3). Such testing is very important as two antibody clones with good kinetic parameters often cannot be paired, for example, if they recognize overlapping epitopes and, in fact, compete for the same binding site. Thus, the proposed here approach based on employment of the label-free mSCI method has enabled a complete cycle of investigation and characterization of the antibodies intended for further development of sandwich-type analytical systems.

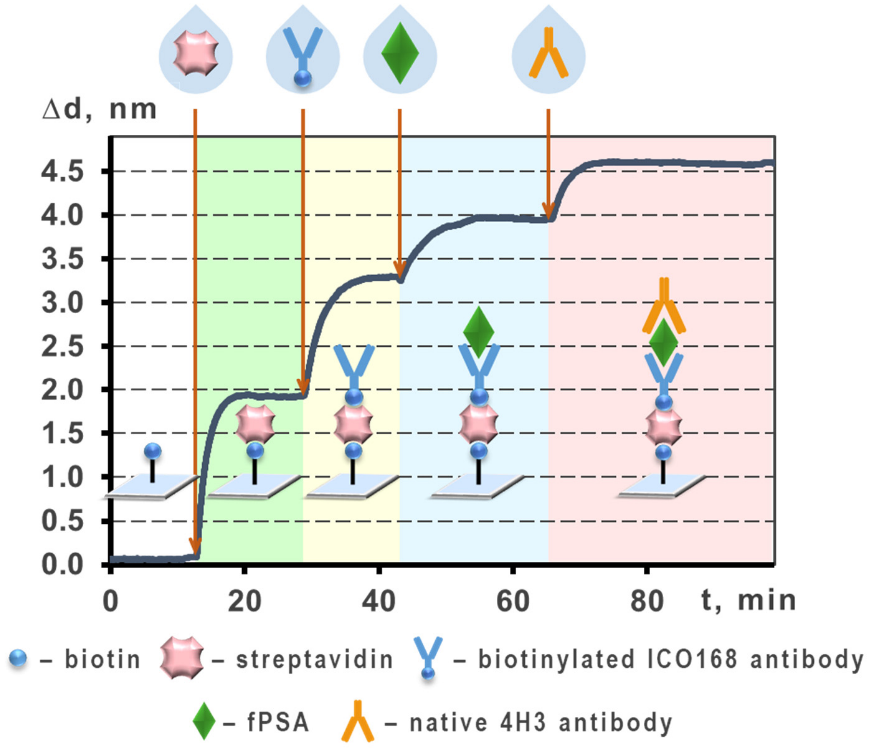


**Figure S3.** Testing epitopes of unlabeled 4H3 and biotinylated ICO168 antibodies for non-overlapping

## S4. Step-by-step characterization of surface modification

The results of step-by-step characterization of surface modification is summarized in Table S1. Eight different types of surface have been studied, each corresponding to one of the modification steps: (1) glass before modification; (2) glass after cleaning with piranha solution (cover glass); (3) cover glass after epoxylation with GLYMO (epoxy glass); (4) epoxy glass after covalent mAb immobilization (functionalized sensor chip); (5) cover glass after amination with APTES (amino glass); (6) amino glass after biotinylation with NHS-biotin (biotinylated glass); (7) biotinylated glass after immobilizaion of streptavidin (streptavidin glass); (8) streptavidin glass after immobilizaion of biotin-mAb (functionalized sensor chip). The water contact angles for all surfaces were measured using a goniometer at the Moscow Institute of Physics and Technology (Moscow, Russia). In the experiments, 5-μL droplets of mQ water were used at room temperature. The mean and standard deviation of five measurements in different areas of the surface are presented in the table.

Meaningful changes of the water contact angle can be seen according to the expected variation in the surface hydrophilicity. Besides, small deviations of the angle values in different areas of the sensor chip indicate homogeneous functionalization over the whole its surface.

**Table S1.** Step-by-step characterization of surface modification

| Step | Contact angle |
|---|---|
| Glass before modification | 71.3 ± 4.2 |
| Glass after cleaning with piranha solution (cover glass) | 26.3 ± 3.8 |
| i.1. Cover glass after epoxylation with GLYMO (epoxy glass) | 38.2 ± 0.8 |
| i.2. Epoxy glass after covalent mAb immobilization (functionalized sensor chip) | 31.2 ± 0.8 |
| ii.1. Cover glass after amination with APTES (amino glass) | 68.7 ± 1.0 |
| ii.2. Amino glass after biotinylation with NHS-biotin (biotinylated glass) | 58.5 ± 2.6 |
| ii.3. Biotinylated glass after immobilizaion of streptavidin (streptavidin glass) | 42.3 ± 4.2 |
| ii.4. Streptavidin glass after immobilizaion of biotin-mAb (functionalized sensor chip) | 30.8 ± 2.3 |

## S5. Long-term stability of magnetic signals

Table S2 shows the magnetic signals recorded immediately and in 2 months after the assay. It can be seen that the signals differ not more than within the error value. That indicates extremely high long-term stability of the magnetic signals.

**Table S2.** Investigation of long-term stability of the registered magnetic signals

| PSA, ng/mL | Mean signal (initial) | Mean signal (after 2 months) |
|---|---|---|
| 0.03 | 92 | 114 |
| 0.1 | 186 | 183 |
| 0.3 | 377 | 359 |
| 1 | 850 | 748 |
| 3 | 1773 | 1801 |
| 7.5 | 3930 | 4127 |
| 15 | 7250 | 7438 |
| 30 | 11770 | 11633 |
| 50 | 17684 | 17845 |
| 75 | 21110 | 20848 |
| 100 | 25790 | 25485 |
